# Epigenetic regulation of ZEB1-RAB25/ESRP1 axis plays a critical role in phenylbutyrate treatment-resistant breast cancer

**DOI:** 10.18632/oncotarget.6480

**Published:** 2015-12-05

**Authors:** Mariko Kikuchi, Keishi Yamashita, Mina Waraya, Naoko Minatani, Hideki Ushiku, Ken Kojo, Akira Ema, Yoshimasa Kosaka, Hiroshi Katoh, Norihiko Sengoku, Takumo Enomoto, Hirokazu Tanino, Masakazu Sawanobori, Masahiko Watanabe

**Affiliations:** ^1^ Department of Surgery, Kitasato University School of Medicine, Kanagawa, Japan; ^2^ Epigenetic Treatment Group, Japan

**Keywords:** phenylbutyrate, breast cancer, epigenetic, histone deacetylase (HDAC), ZEB1

## Abstract

Phenylbutyrate (PB) is a histone deacetylase antagonist that also exhibits antitumor activity. In this study, we used 7 breast cancer cell lines to identify biomarker candidates that predict PB sensitivity in breast cancer.

Comprehensive gene expression profiles were compared using microarrays, and the importance of the identified genes to PB sensitivity was confirmed in gene transfection experiments. CRL and MDAMB453 cells were identified as PB-sensitive, while MDAMB231 cells were PB-resistant.RAB25 and ESRP1 were identified as key regulators of PB sensitivity, while ANKD1, ETS1, PTRF, IFI16 and KIAA1199 acted as PB resistance-related genes. Expression of these genes was dramatically altered by DNA demethylation treatments. RAB25 expression inhibited IFI16 and PTRF, while ESRP1 expression suppressed ANKRD1, ETS1, and KIAA1199. Both RAB25 and ESRP1 were suppressed by ZEB1, which was in turn regulated via epigenetic mechanisms. Thus, PB sensitivity is influenced by epigenetic expression alteration of ZEB1. The genes associated with PB sensitivity are downstream targets of ZEB1. Epigenetic regulation of ZEB1 may prove valuable as a critical biomarker for predicting resistance to breast cancer therapies.

## INTRODUCTION

Phenylbutyrate (PB) has been used to treat children with hyperammonemia resulting from an inherited urea cycle abnormality [1]. PB causes the excretion of glutamine in urine, which decreases blood ammonia levels [2]. In addition, PB is a histone deacetylase (HDAC) antagonist that also exhibits anticancer effects *in vitro* [3]. A preclinical study showed that PB has cytotoxic effects at concentrations greater than 0.5 mM [4]. A phase I clinical trial recommended a dosage of 27 g/day for patients with solid tumors, as the blood PB concentration reached 0.5-3 mM under that regimen [4] Significant clinical anticancer effects were reported for leukemia [5-8], colorectal cancer [9] and prostatic cancer [10]. However, no reports have yet described the clinical efficacy of PB for treating breast cancer.

Breast cancer is the most prevalent cancer among females in Europe and the United States [11]. Breast cancer is also the most prevalent carcinoma in Japanese women, ranking as the fifth leading cause of death among females, despite the nation-wide spread of surveillance systems and the emergence of novel anticancer drugs [12]. Progress in hormone therapy, chemotherapy, and molecular therapies has dramatically improved clinical outcomes for breast cancer. However, resistance to these therapies is a major obstacle to breast cancer treatment [13-15], and the molecular mechanisms underlying resistance remain largely unknown.

In this study, we clarified the effects of PB in breast cancer and identified PB-sensitive breast cancer cell lines. We also investigated gene expression profiles to identify biomarkers predictive of PB sensitivity.

## RESULTS

### Selection of PB-sensitive and PB-resistant breast cancer cell lines

**Figure 1:**
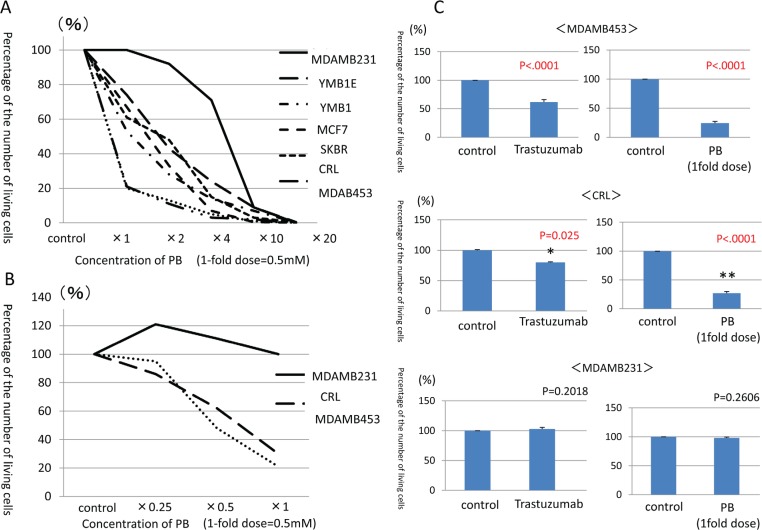
Counts of viable breast cancer cells after administration of PB and trastuzumab **A**. Counts of viable cells after 7 days of PB administration compared to control cells. **B**. Counts of viable cells after administration of lower PB doses (0.5-fold dosage at 0.25mM and 0.25-fold dosage at 0.125mM) in PB-sensitive cells as compared to PB-resistant cells. **C**. Counts of viable cells after PB administration compared to trastuzumab administration.

Cell proliferation was assessed in seven breast cancer cell lines following PB treatments. Viable cells were counted on day 7 and compared to control cell counts (Fig. 1A). MDAMB453 and CRL cell counts were reduced by 70-80% at the 1-fold PB dose in comparison with the control cells. SKBR, MCF7, YMB1E, YMB1 cell counts were decreased by 30% at the 1-fold PB dose, and decreased by 80% at the 4-fold PB dose. Only MDAMB231 cell counts did not decrease at all at either the 1-fold or 2-fold PB dosage, but they did decrease by 80% at the 10-fold PB dosage. These findings suggested that, although cell proliferation could be suppressed by PB treatment in all seven lines, differences in gene expression confer different sensitivities to PB depending on breast cancer cell type. Therefore, MDAMB453 and CRL cells were designated PB-sensitive strains, while MDAMB231 cells were designated a PB-resistant strain.

The effects of the 1-fold PB dosage on each breast cancer cell line are shown in Table 1. Sensitive cell lines were Her2-positive (CRL and MDAMB453), while the resistant cell line was Triple Negative (TN, MDAMB231). In the sensitive cell lines (MDAMB453 and CRL cells), PB reduced proliferation in a dose-dependent manner even at lower doses (0.5-fold at 0.25 mM and 0.25-fold at 0.125 mM)these lower PB dosages had no effect in the resistant (MDAMB231) cells (Fig. 1B). We then compared the effect of PB with that of Trastuzumab, which decreases proliferation of Her2-positive cells both *in vitro* and *in vivo*, in the Her2-positive, PB-sensitive cell lines [16, 17]. PB reduced proliferation much more than 10μg/ml Trastuzumab in both the MDAMB453 and CRL cell lines, while neither PB nor Trastuzumab reduced MDAMB231 cell proliferation (Fig. 1C).

**Table 1:** Comparison of reduction rate by PB treatment and subtype

	Number of cells in control(/ml)	Number of cells after PB treatment(/ml)	Reduction rate(%)	Subtype
MDAMB453	4.3×10^6^	8.7×10^5^	−80%	HER2
CRL	2.9×10^6^	6.1×10^5^	−71%	HER2
SKBR	3.1×10^6^	1.9×10^6^	−39%	HER2
MCF7	4.5×10^6^	3.0×10^6^	−34%	luminalB
YMB1E	4.7×10^6^	2.5×10^6^	−30%	HER2
YMB1	2.3×10^6^	1.7×10^6^	−26%	HER2
MDAMB231	1.2×10^6^	1.2×10^6^	0%	TN

### Identification of genes related to PB sensitivity and resistance using expression microarrays and semi-quantitative RT-PCR

**Figure 2:**
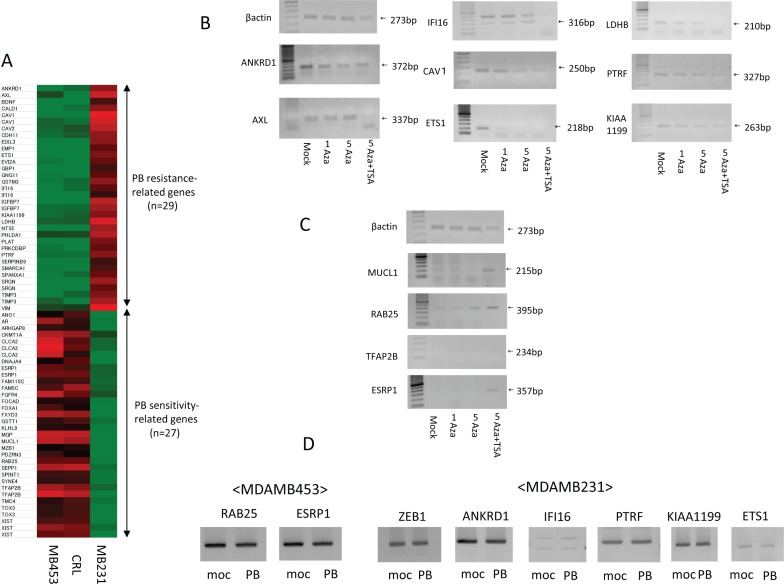
Identification of PB sensitivity-related genes and PB resistance-related genes using expression microarrays and expression changes after demethylation treatment **A**. Heat map of Affymetrix gene expression in PB-resistant breast cancer strains compared to PB-sensitive breast cancer strains. **B**. Demethylation treatment decreases expression of PB resistance-related genes in MDAMB231 cells. **C**. Demethylation treatment increases expression of PB sensitivity -related genes in MDAMB231 cells. **D**. Expression of ZEB1 and its downstream genes in PB treated and untreated cells.

To explore the molecular profiles underlying PB sensitivity, expression microarrays were performed. Heat maps of comparative gene expression generated using Affymetrix are shown for PB-resistant and PB-sensitive strains (Fig. 2A). The top 29 genes highly expressed in PB-resistant strains and minimally expressed in PB-sensitive strains were designated PB resistance-related genes (Fig. S1A). These genes included ANKLD1, AXL, CAV1/2, LDHB, ETS1, IFI16, PTRF (cavin), KIAA1199 and VIM, which have previously been linked to drug resistance in breast and other cancers [18-28].

The PB sensitivity-related genes (the top 27 genes highly expressed in PB-sensitive strains and minimally expressed in PB-resistant strains) identified in the microarray study included CLCA2, ESRP1, FGFR4, MUCL1, FXYD3, RAB25, SEPP1, TFAP2B, and TOX3 (Fig. S1B). Reports of reduced expression and hypermethylation of promoter regions of these genes in human cancer tissues [29-38] suggests that they have tumor suppressor activity.

We confirmed that mRNA expression of those PB restistance-related genes was low in MDAMB453 and CRL cells, and high in MDAMB231 cells, using semi-quantitative RT-PCR (Fig. S1C). High mRNA expression of PB sensitivity-related genes in MDAMB453 and CRL cells, and low expression in MDAMB231 cells, was also confirmed using this method (Fig. S1D). RT-PCR analysis recapitulated the microarray results, indicating that Affymetrix expression microarray results were reliable.

### Demethylation treatment changes expression of PB resistance and sensitivity-related genes

Simultaneous treatment with 5 Aza-dC and the HDAC inhibitor TSA, a highly effective demethylation method [39], was used to evaluate whether epigenetic factors affect the differential expression of the genes identified above [40, 41].

Expression of eight PB resistance-related genes decreased in the PB-resistant MDAMB231 cells after demethylation treatment (Fig. 2B).

Conversely, expression of four PB sensitivity-related genes increased after demethylation treatment in MDAMB231 cells (Fig. 2C). The combination of 5-Aza-dC and TSA primarily reactivates target genes, so PB sensitivity-related genes were considered sensitive to primary upstream alterations. We focused on specifically on MUCL1, RAB25, TFAP2B, and ESRP1 in subsequent experiments.

### Expression of ZEB1 and its pathway genes in PB treated and untreated cells

We confirmed expression of ZEB1 and its pathway genes in PB (1-fold dose) treated and untreated cells. In MDAMB453 cells, expression of PB sensitivity-related genes (RAB25, ESRP1) did not change. Similarly, in MDAMB231 cells, expression of PB resistance-related genes (ZEB1, ANKRD1, IFI16, PTRF, KIAA1199, and ETS1) did not change (Fig. 2D).

### Relationship of resistance-associated genes to sensitivity-related genes

**Figure 3:**
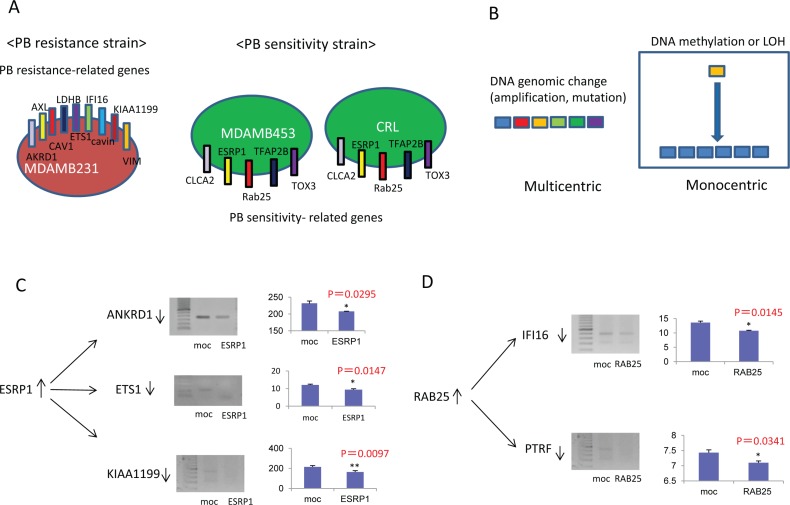
Relationship between resistance-related and sensitivity-related genes **A**. Differential gene expression in PB-resistant and PB-sensitive cell lines. **B**. Molecular regulation of PB sensitivity be monocentrically dependent on epigenetic regulation rather than multicentrically dependent on a variety of genes. **C**. Transfection of the ESRP1 gene into PB-resistant MDAMB231 cells. **D**. Transfection of the RAB25 gene into PB-resistant MDAMB231 cells.

Differential gene expression between PB-resistant and PB-sensitive cell lines suggested that alterations in molecular mechanisms affected by these genes might explain the phenotypic difference (Fig. 3A). Possible mechanisms in cancer cells include genomic and/or epigenetic changes. If epigenetic changes drive these expression differences, demethylation treatment should reactivate PB sensitivity-related genes and silence PB resistance-delated genes. Importantly, the molecular alterations associated with PB sensitivity are likely to be monocentric by epigenetic mechanism (Fig. 3B).

### Transfection of PB sensitivity-related genes into PB-resistant MDAMB231 cells

#### Transient transfection of a plasmid vector the expressing

full-length ESRP1 gene into MDAMB231 cells reduced expression of the PB resistance-related genes ANKRD1, ETS1, and KIAA1199 (p=0.0295, p=0.0147, and p=0.0097, respectively), suggesting that ESRP1 is an upstream regulator of ANKRD1, ETS1, and KIAA1199 (Fig. 3C). Similarly, transient transfection of a RAB25-expressing vector into MDAMB231 cells reduced expression of the PB resistance-related genes IFI16 and PTRF (p=0.0145 and p=0.0341, respectively), suggesting that RAB25 is an upstream regulator of IFI16 and PTRF (Fig. 3D).

Transient transfection with plasmids containing either full-length MUCL1 or TFAP2B into MDAMB231 cells did not alter the expression of PB resistance-related genes (data not shown). Together, these findings indicate that ESRP1 and RAB25 may play a critical and separate roles in drug resistance by affection the expression of ANKRD1, ETS1, KIAA1199, IFI16, and PTRF.

**Figure 4:**
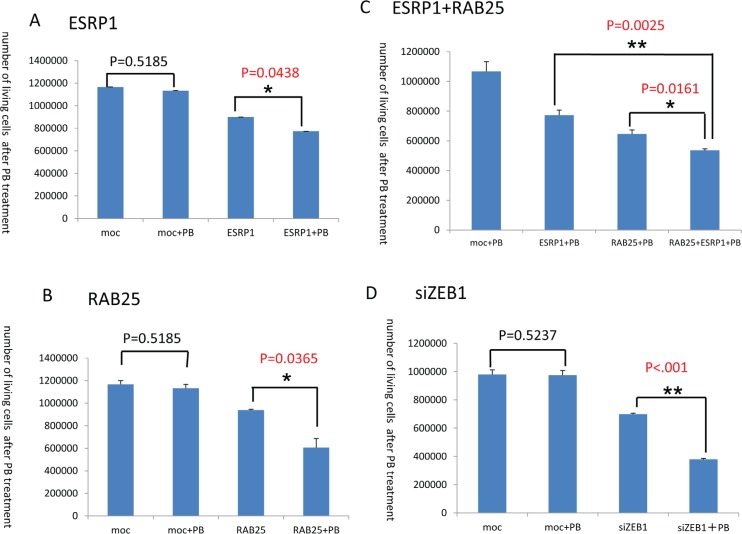
PB administration decreases cell proliferation **A**. Counts of MDAMB231 cells transiently transfected with the ESRP1 gene after PB administration. **B**. Counts of MDAMB231 cells transiently transfected with the RAB25 gene after PB administration. **C**. Counts of MDAMB231 cells transiently transfected with both the ESRP1 and RAB25 genes after PB administration. **D**. Counts of MDAMB231 cells after ZEB1 gene knockdown via transient transfection of ZEB1 siRNA and PB administration.

In addition, administration of the 1-fold PB dosage to MDAMB231 cells transiently transfected with the ESRP1 gene decreased cell counts (p=0.0438) (Fig. 4A). Similarly, administration of the same PB dosage to the MDAMB231 cells transiently transfected with the RAB25 gene also decreased cell counts (p=0.0365) (Fig. 4B). Moreover, exposure to the 1-fold PB dosage decreased counts in MDAMB231 cells transiently transfected with both the ESRP1 and RAB25 genes even further (p=0.0025, p=0.0161) (Fig. 4C). Thus, ESRP1 and RAB25 independently and synergistically suppressed cell proliferation following PB administration.

### ESRP1 and RAB25 were not directly regulated by promoter DNA methylation in breast cancer

#### Expression of ESRP1 and RAB25 was low in MDAMB231

cells, and demethylation treatments of 5 Aza-dC and trichostatin A robustly increased expression of these two genes (Fig. 2C). Hypermethylation of the DNA promoter region for RAB25 has been reported previously, and both RAB25 and ESRP1 have tumor-suppressive effects [35]. Regulation of the expression of these two genes via promoter methylation might also affect proliferation in human cancers. However, we did not find CpG islands in the promoter DNA for RAB25, and no methylation in the promoter region for ESRP1 was detected by direct sequencing (data not shown). These findings suggested that neither gene is regulated directly by promoter DNA methylation, but their expression may be indirectly regulated by other methylation-sensitive genes. Both RAB25 and ESRP1 are strongly downregulated by the expression of ZEB1, the epithelial-mesenchymal transition (EMT) inducer, in a Tetracycline induced model of human cancer [42].

### Dyregulation of ZEB1 alters RAB25/ESRP1 expression in breast cancer cell lines

**Figure 5:**
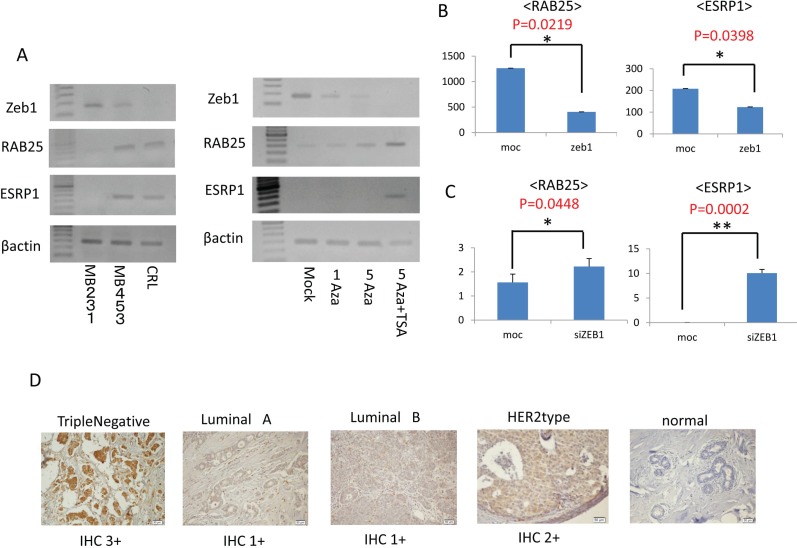
Zeb1 expression and immunohistochemistry **A**. Left panel: Expression of ZEB1 mRNA in MDAMB231, MDAMB453 and CRL cells measured using RT-PCR. Right panel: Change in expression of ZEB1 mRNA after demethylation treatment measured using RT-PCR. **B**. Expression of the RAB25 and ESRP1 genes in MDAMB453 cells after transfection with the ZEB1 gene. **C**. Expression of the RAB25 and ESRP1 genes in MDAMB231 cells after ZEB1 knockdown resulting from transient transfection of ZEB1 siRNA. **D**. Immunohistochemistry for ZEB1 in breast cancer tissues and normal breast tissues.

In our microarray experiment, ZEB1 was expressed only in MDAMB231 cells and not in MDAMB453 and CRL cells. The expression of ZEB1 was confirmed by measuring mRNA levels in MDAMB231, MDAMB453 and CRL cells using RT-PCR (left panel, Fig. 5A). Expression of ZEB1 was high in MDAMB231 cells and low in MDAMB453 and CRL cells. Interestingly, levels of both RAB25 and ESRP1 mRNA were high when ZEB1 mRNA levels were low and vice versa, which is consistent with a previous report examining other cancers [42]. These reciprocal relations between ZEB1 and RAB25/ESRP1 were also observed in MDAMB231 cells following demethylation treatments (right panel, Fig. 5A). These findings suggest that ZEB1 regulates RAB25/ESRP1 expression at mRNA level in human cancer.

To investigate this directly, expression of the RAB25 and ESRP1 genes was quantified in MDAMB453 cells transfected with ZEB1 using Q-RT-PCR (Fig. 5B). Both RAB25 and ESRP1 were suppressed in MDAMB453 cells after transient transfection of ZEB1. Conversely, expression of both RAB25 and ESRP1 was activated in MDAMB231 cells after ZEB1 knockdown by transient siRNA transfection (Fig. 5C). ZEB1 knockdown in MDAMB231 cells was synergistically suppressed by concurrent administration of PB solution 1x as expected (Fig. 4D).

### Immunohistochemistry

ZEB1 protein levels were rated IHC 1+ in 15 breast cancer samples, IHC 2+ in 16 samples, and IHC 3+ in 5 samples (Fig. 5D). ZEB1 was more strongly expressed in TN breast cancer than in the other cancers (p<.0001) (Table 2). Normal breast tissue did not show any ZEB1 staining.

**Table 2:** Zeb1 Immunohistochemistry

		n(%)	IHC 1+	IHC 2+	IHC 3+	p-value
Histological type	Papillotubular Ca.	16(40)	6	8	2	0.4782
	Solid-tublar Ca.	24(60)	14	6	4	
Stage	I	13(33)	7	4	2	0.4898
	II	25(63)	11	10	4	
	III	2(4)	2	0	0	
Subtype	luminalA	12(30)	6	6	0	<. 0001
	luminalB	11(28)	9	2	0	
	HER2type	5(12)	5	0	0	
	TN	12(30)	0	6	6	

### Expression of ZEB1 pathway genes in breast cancer clinical materials

**Figure 6:**
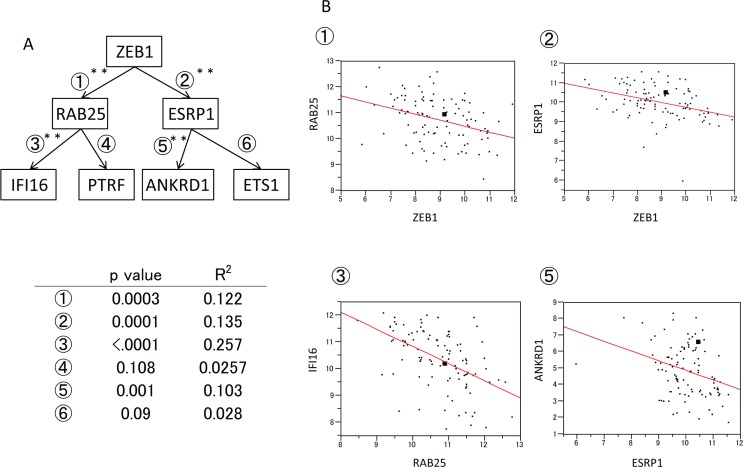
Expression of ZEB1 pathway genes in clinical breast cancer materials **A**. Expression of ZEB1 pathway genes in breast cancer samples determined using microarrays. **B**. Diagram of correlations between the expression of ①ZEB1 and RAB25, ②ZEB1 and ESRP1, ③RAB25 and IFI16, and ⑤ESRP1 and ANKRD1.

Significant inverse correlations were found between ZEB1 and RAB25, ZEB1 and ESRP1, RAB25 and IFI16, RAB25 and PTRF, ESRP1 and ANKRD1, and ESRP1 and ETS1 (Fig. 6A). This was consistent with the cell line data in our current study. The correlations between ZEB1 and RAB25, ZEB1 and ESRP1, RAB25 and IFI16, and ESRP1 and ANKRD1 were particularly strong (Fig. 6B).

## DISCUSSION

In this study, we demonstrated that PB is an effective inhibitor of cell proliferation in several breast cancer cell lines. In fact, PB suppressed HER2-positive cell proliferation more effectively than the HER2 antibody trastuzumab. However, proliferation of TN-positive breast cancer cells was not affected by PB. The search for differences in gene expression that might contribute to PB resistance suggested a role for the ZEB1-RAB25/ESRP1 pathway, which is involved in epithelial-mesenchymal transition (EMT) and is known to affect chemoresistance in breast cancer.

EMT, and the associated stem cell-like phenotype, is considered a major cause of resistance to therapy in breast cancer [43, 44]. The EMT activator ZEB1, in particular, has been shown to confer stemness and resistance to treatment [45]. Levels of ZEB1 protein are reduced by the miR-200 microRNA family, and aberrant epigenetic silencing of miR-200 in human cancer cells and tumors promotes EMT, tumor cell adhesion, migration, invasion and metastasis [46]. Overexpression of ZEB1 also downregulates miR-200 and is associated with a pro-survival and drug-resistant phenotype [46, 47].

In the present study, 8 genes (ANKLD1, AXL, CAV1, ETS1, IFI16, LDHB, PTRF (cavin) and KIAA1199) were identified as PB resistance-related, and the expression of most of them was controlled by RAB25 or ESRP1. Although expression of PB resistance-related genes was downregulated by demethylation treatments, there have been no reports of genomic amplification for these genes, suggesting that epigenetic changes alter their expression indirectly via upstream molecules. Thus RAB25 and ESRP1 likely play a central role in dynamic changes in the expression of PB resistance-related genes.

Most of the PB resistance-related genes identified here have established effects on chemoresistance. ANKRD1 is a novel determinant of cisplatin resistance in ovarian and breast cancers [18]. AXL belongs to a tyrosine kinase family, and its inhibition promotes apoptosis, blocks growth and enhances chemosensitivity in human non-small cell lung cancer [19]. IFI16 (IFN-gamma-inducible protein 16) epigenetically suppresses estrogen receptor expression, and it may affect tamoxifen sensitivity in breast cancer cells [25]. CAV1 is associated with sensitivity to cisplatin and anthracyclin [20, 21], while ETS1 is associated with both sensitivity to gemcitabine and multidrug resistance (MDR) [23]. Polymerase I and transcript release factor (PTRF, cavin-1), which is a component of caveolae along with CAV1, is associated with chemoresistance in glioblastoma [26]. KIAA1199 expression, which has been localized to the endoplasmic reticulum, has not yet been linked to drug resistance, but it does affect malignant potential in breast cancer [27]. All of these genes were suppressed by demethylation treatment, and upstream epigenetic targets are likely important in controlling their expression.

Because such upstream gene candidates must be reactivated through demethylation, we focused on RAB25, ESRP1, TFAP2B and MUCL1. Among these four candidates, only RAB25 and ESRP1 affected expression of PB resistance-related genes. Neither TFAP2B nor MUCL1 altered PB resistance-related gene mRNA levels (data not shown). Interestingly, levels of each mRNA were downregulated by ESRP1 or RAB25, but not by the two together. ESRP1 suppressed ANKD1, ETS1 and KIAA1199, while RAB25 suppressed IFI16 and PTRF. This suggests ESRP1 and RAB25 are independently involved in PB drug sensitivity, and the two act synergistically to suppress PB-resistant cell proliferation.

After transfection with RAB25, MDAMB231 cell proliferation was dramatically reduced. RAB25 is a known tumor suppressor gene, acting through multiple pathways to enhance apoptosis and suppress angiogenesis and invasion by modulating VEGF-A and VEGFR-1 expression [35]. ESRP1 (epithelial splicing regulatory protein 1), which regulates alternative splicing events in epithelial cells, also has tumor suppressor effects and is down-regulated during EMT [31]. Both RAB25 and ESRP1 were robustly reactivated by demethylation treatment, but no evidence of promoter DNA methylation was found in either gene.

It is possible that both genes are regulated by other cancer-relevant genes that are affected by promoter DNA methylation. ZEB1 is one candidate that may affect breast cancer in this way. ZEB1 levels are controlled by miR-200 family microRNAs, the expression of which is also influenced by DNA methylation in human cancer. Decreased expression of miR-200 by DNA methylation promotes ZEB1 expression [48, 49], and ZEB1 is strongly suppressed by demethylation treatment. Reducing ZEB1 expression increases levels of ESRP1 and RAB25 in human cancer and vice versa, and in our study ZEB1 directly affected transcription of both genes. Most intriguingly, ESRP1 and RAB25 synergistically suppressed proliferation of PB-resistant, MDAMB231 (TN) cells, and siRNA-induced knockdown of ZEB1 reduced proliferation even more. This suggests ZEB1 expression may play a crucial role in PB resistance in TN breast cancers. Immunohistochemistry confirmed that ZEB1 is strongly expressed in TN breast cancer, as compared to other cancers. Together, these findings support the idea of using epigenetic suppression of ZEB1 as a novel therapeutic strategy to help overcome drug resistance in human breast cancer.

In cell lines and primary breast cancer tissue, ZEB1 reduces the expression of Both RAB25 and ESRP1, which have tumor suppressor functions. RAB25 induces apoptosis in TN breast cancer [50], and reduced expression of RAB25 is involved in PB resistance. ESRP1 inhibits EMT in breast cancer by antagonizing hnRNPM [51]. Here, we provide evidence that RAB25 affects the expression of IFI16 and PTRF, while ESRP affects the expression of ANKD1, ETS1 and KIAA1199. Of these, expression of IFI16 and ANKD1 were particularly strongly associated with RAB25 and ESRP1 levels in primary breast cancer. Expression of IFI16 plays a role in resistance to tamoxifen treatment in breast cancer [25], and ANKD1 is involved in resistance to cisplatin resistant in ovarian and breast cancers [18]. Thus, the ZEB1 pathway affects expression of a variety of genes that play important roles in drug resistance during cancer treatment.

## CONCLUSIONS

Our findings show that PB sensitivity is controlled through epigenetic regulation of a ZEB1-RAB25/ESRP1 pathway, and several candidate genes associated with PB resistance downstream of ZEB1 have been identified. Epigenetic regulation of ZEB1 may serve as a critical biomarker for predicting resistance to breast cancer treatments.

## MATERIALS AND METHODS

### Cell lines

We used 7 breast cancer (BC) cell lines (MDAMB231, CRL, SKBR, YMB1, YMB1E, MDAMB453, and MCF7). MDAMB231, CRL, SKBR, and YMB1 cells were kindly provided by the Kyushu University Beppu Hospital (Oita, Japan). YMB1E cells were provided by the Cell Resource

Center of the Biomedical Research Institute of Development and Aging, Tohoku University (Sendai, Japan). MDAMB453 and MCF7 cells were purchased from RIKEN Bio Resource Centre (Ibaraki, Japan). The CRL, SKBR, YMB1, YMB1E, and MCF7 cells were maintained in RPMI1640 Medium (GIBCO, Carlsbad, CA), the MDAMB453 cells were maintained in L15 Medium(GIBCO), and the MDAMB231 cells were maintained in DMEM Medium (Sigma Aldrich, St Louis, MO, USA). All media contained 10% fetal bovine serum (FBS) and Penicillin-Streptomycin (GIBCO).

### PB and trastuzumab treatments in breast cancer cell lines

1×10^6^ cells from each cell line were incubated in PB for 24 h. One tablet of PB (1g, triButyrate®, Fyrklövern Scandinavia AB, Sweden) was dissolved in 100 mL distilled water, and the PB solution was filtered using a Corning® 250 mL Vacuum Filter System (0.22μm Pore) (Corning, Tewksbury, MA). Previous literature demonstrated that serum PB concentrations could reach 0.5 mM-3 mM in humans when administered at dosage of 27 or 36 g/ day [4]. We thus designated 0.5mM as 1-fold PB dosage, and 2-fold (1 mM), 4-fold (2 mM), 10-fold (5 mM), and 20-fold (10 mM) dosages of PB were also used in this study. Cell growth was observed for 7 days, during which the culture medium and PB solution were replaced every 72 h. Controls were not exposed to the PB solution. On day 7, cells were detached using Trypsin-EDTA (GIBCO) and viable cells were counted using a Countess automated cell counter (Invitrogen Life Technologies, Carlsbad, CA). Finally, an additional 1×10^6^ cells from each cell line were treated in the same manner with trastuzumab (10μg/ml) (Roche, Basel, Schweizerland) rather than PB, and viable cells were also counted on day 7.

### 5-aza-2′-deoxycytidine (5 Aza-dC) and trichostatin A (TSA) treatments in BC cell lines

Cells from the PB-resistant strain (1×10^6^ cells/T-75 flask) were treated with 1 or 5μM of the demethylating agent 5-aza-2′-deoxycytidine (5 Aza-dC) (Sigma-Aldrich) dissolved in 50% acetic acid (Wako pure Chemical Industries, Osaka, Japan) once every 24h for 4 days. Controls were mock-treatment with PBS (Phosphate Buffered Saline, GIBCO) in the same amount of acetic acid once every 24 h for 4 days. In addition, 300 nM of the HDAC inhibitor trichostatin A (TSA) (Sigma-Aldrich) was added to the medium for the final 24 h.

### Expression microarrays

mRNA was extracted from the PB-sensitive strains (CRL and MDAMB453 cells) and the PB-resistant strain (MDAMB231 cell), we extracted mRNA using an RNeasy Mini Kit (QIAGEN Sciences, Maryland, USA). Gene profiles were compared using Affymetrix 3′ IVT Express Kit microarrays (harboring 54,675 genes) according to the manufacturer's instructions. Genes expressed at high levels in the PB-sensitive strains and at low levels in the PB-resistant strain were categorized as sensitivity-related genes. Similarly, PB resistance-related genes were expressed at high levels in the PB-resistant strain and at low levels in the PB-sensitive strains.

### Semi-quantitative reverse transcription polymerase chain reaction (RT-PCR) and quantitative RT-PCR (Q-RT-PCR)

Total RNA was extracted from all cell lines using a RNeasy Mini Kit (QIAGEN) and reverse-transcribed using a SuperScript III reverse transcriptase kit (Invitrogen). Quantitative RT-PCR (Q-RT-PCR) was conducted in triplicate using iQ Supermix (Bio-Rad) on the C1000 Touch TM Thermal Cycler CFX96 Real Time System (Bio-Rad). PCR conditions and primer sequences are provided in Table S1.

### Plasmid and transfection

Full-length cDNA sequences of the MUCL1 and RAB25 genes were isolated by PCR and sub-cloned into a pcDNA^TM^3.1D/V5-His-TOPO vector (Invitrogen). The vector with self-ligation was used as a control. ESRP1, TFAP2, ZEB1, and control plasmids were purchased from OriGene Technologies (Rockville, MD, USA). Cells were seeded in 10cm dishes overnight to reach 90-95% confluence. Then they were transfected with 24μg of plasmid vector using Lipofectamine 2000 reagent (Invitrogen) in OPTI-MEM medium (GIBCO). Six hours later, the transfected cells were transferred to complete medium. After 48 h, the cells were harvested and used for RT-PCR and Q-RT-PCR.

### siRNA and transfection

Small-interfering RNA (siRNA) targeting human ZEB1 was purchased from Sigma Aldrich (St Louis, MO, USA). The ZEB1 siRNA sequences were as follows: CCAAUAAGCAAACGAUUCUGA, antisense: AGAAUCGUUUGCUUAUUGGCA. We used MISSION siRNA Universal Negative Control (Sigma Aldrich) as a control. Cells were seeded in 10cm dishes overnight to reach 30-40% confluence. Then they were transfected with 600 pmol of siRNA using Lipofectamine 2000 reagent (Invitrogen) in OPTI-MEM medium (GIBCO). Six hours later, the transfected cells were transferred to complete medium. After 48 h, the cells were harvested and used for RT-PCR and Q-RT-PCR.

### Immunohistochemistry

We recruited 40 primary BC patients (Triple Negative, TN = 12 cases, luminal A = 12 cases, luminal B = 10 cases, HER2 type = 6 cases) with no prior chemotherapy who underwent surgical resection of the primary tumors at Kitasato University Hospital between January 1, 1996 and December 31, 2000. All patients agreed to the use of pathological specimens. Tissues were fixed in formalin and embedded in paraffin, and 4 μm thick serial sections were used. The slices were incubated with 3 % H_2_O_2_ at room temperature for 5 min to deactivate endogenous peroxidase and then washed with PBS. Rabbit anti-human ZEB1 polyclonal antibody (Atlas Antibodies, Stockholm, Sweden; 1:50 dilution) was added and the slices were incubated at 4°C overnight. Immune complexes were amplified using a Vectastain Elite ABC kit (Vector Laboratories, Inc, Burlingame, CA) according to the manufacturer's instructions. These complexes were then detected by incubating with the chromogen 3,3′-diaminobenzidine (DAB) substrate for 5 minutes. ZEB1 expression levels were categorized according to the diagnostic criteria of American Society of Clinical Oncology/College of American Pathology 2007 guidelines as follows [52]: IHC 1+: light staining of more than 10% of the specimen; IHC 2+: moderate staining of more than 10% and less than or equal to 30% of the specimen; IHC 3+: strong staining of more than 30% of the specimen.

### Expression of ZEB1 pathway genes in breast cancer clinical samples

Microarray data from the MD Anderson Cancer Center (Institut Goustave Russy cohort, GDS4057, N=103) were downloaded from GEO

(http://www.ncbi.nlm.nih.gov/geo/query/acc.cgi acc.GDS4057).

Expression levels of ①ZEB1/RAB25, 2ZEB1/ESRP1, 3RAB25/IFI16,

④RAB25/PTRF, ⑤ESRP1/ANKRD1, and ⑥ESRP1/ETS1 were plotted. Note that KIAA1199 levels were not measured in this microarray.

## SUPPLEMENTARY MATERIAL TABLE AND FIGURE



## References

[1] Walker V (2014). Ammonia metabolism and hyperammonemic disorders. Adv Clin Chem.

[2] Cordoba J, Ventura-Cots M (2014). Drug-induced removal of nitrogen derivatives in urine: a new concept whose time has come. Hepatology.

[3] Kouraklis G, Theocharis S (2002). Histone deacetylase inhibitors and anticancer therapy. Curr Med Chem Anticancer Agents.

[4] Gilbert J, Baker SD, Bowling MK, Grochow L, Figg WD, Zabelina Y, Donehower RC, Carducci MA (2001). A phase I dose escalation and bioavailability study of oral sodium phenylbutyrate in patients with refractory solid tumor malignancies. Clin Cancer Res.

[5] Gore SD, Samid D, Weng LJ (1997). Impact of the putative differentiating agents sodium phenylbutyrate and sodium phenylacetate on proliferation, differentiation, and apoptosis of primary neoplastic myeloid cells. Clin Cancer Res.

[6] Witzig TE, Timm M, Stenson M, Svingen PA, Kaufmann SH (2000). Induction of apoptosis in malignant B cells by phenylbutyrate or phenylacetate in combination with chemotherapeutic agents. Clin Cancer Res.

[7] Gore SD, Baylin S, Sugar E, Carraway H, Miller CB, Carducci M, Grever M, Galm O, Dauses T, Karp JE, Rudek MA, Zhao M, Smith BD (2006). Combined DNA methyltransferase and histone deacetylase inhibition in the treatment of myeloid neoplasms. Cancer Res.

[8] Maslak P, Chanel S, Camacho LH, Soignet S, Pandolfi PP, Guernah I, Warrell R, Nimer S (2006). Pilot study of combination transcriptional modulation therapy with sodium phenylbutyrate and 5-azacytidine in patients with acute myeloid leukemia or myelodysplastic syndrome. Leukemia.

[9] Camacho LH, Olson J, Tong WP, Young CW, Spriggs DR, Malkin MG (2007). Phase I dose escalation clinical trial of phenylbutyrate sodium administered twice daily to patients with advanced solid tumors. Invest New Drugs.

[10] Kulp SK, Chen CS, Wang DS, Chen CY, Chen CS (2006). Antitumor effects of a novel phenylbutyrate-based histone deacetylase inhibitor, (S)-HDAC-42, in prostate cancer. Clin Cancer Res.

[11] Miller E, Lee HJ, Lulla A, Hernandez L, Gokare P, Lim B (2014). Current treatment of early breast cancer: adjuvant and neoadjuvant therapy. F1000Res.

[12] Uchida K, Ohashi H, Kinoshita S, Nogi H, Kato K, Toriumi Y, Yamashita A, Kamio M, Mimoto R, Takeyama H (2013). Breast cancer screening and the changing population pyramid of Japan. Breast Cancer.

[13] Ravdin PM (1995). Anthracycline resistance in breast cancer: clinical applications of current knowledge. Eur J Cancer.

[14] Goss P (2002). Anti-aromatase agents in the treatment and prevention of breast cancer. Cancer Control.

[15] O'Driscoll L, Clynes M (2006). Biomarkers and multiple drug resistance in breast cancer. Curr Cancer Drug Targets.

[16] Tahmasebi F, Kazemi T, Amiri MM, Khoshnoodi J, Mahmoudian J, Bayat AA, Jeddi-Tehrani M, Rabbani H, Shokri F (2012). In vitro assessment of the effects of anti-HER2 monoclonal antibodies on proliferation of HER2-overexpressing breast cancer cells. Immunotherapy.

[17] Spicer J, Harries M, Ellis P (2005). Adjuvant trastuzumab for HER2-positive breast cancer. Lancet.

[18] Scurr LL, Guminski AD, Chiew YE, Balleine RL, Sharma R, Lei Y, Pryor K, Wain GV, Brand A, Byth K, Kennedy C, Rizos H, Harnett PR (2008). Ankyrin repeat domain 1, ANKRD1, a novel determinant of cisplatin sensitivity expressed in ovarian cancer. Clin Cancer Res.

[19] Linger RM, Cohen RA, Cummings CT, Sather S, Migdall-Wilson J, Middleton DH, Lu X, Baron AE, Franklin WA, Merrick DT, Jedlicka P, DeRyckere D, Heasley LE (2012). Mer or Axl receptor tyrosine kinase inhibition promotes apoptosis, blocks growth and enhances chemosensitivity of human non-small cell lung cancer. Oncogene.

[20] Nakatani K, Wada T, Nakamura M, Uzawa K, Tanzawa H, Fujita S (2005). Expression of caveolin-1 and its correlation with cisplatin sensitivity in oral squamous cell carcinoma. J Cancer Res Clin Oncol.

[21] Yuan G, Regel I, Lian F, Friedrich T, Hitkova I, Hofheinz RD, Strobel P, Langer R, Keller G, Rocken C, Zimmermann W, Schmid RM, Ebert MP (2013). WNT6 is a novel target gene of caveolin-1 promoting chemoresistance to epirubicin in human gastric cancer cells. Oncogene.

[22] McCleland ML, Adler AS, Shang Y, Hunsaker T, Truong T, Peterson D, Torres E, Li L, Haley B, Stephan JP, Belvin M, Hatzivassiliou G, Blackwood EM (2012). An integrated genomic screen identifies LDHB as an essential gene for triple-negative breast cancer. Cancer Res.

[23] Kars MD, Iseri OD, Gunduz U (2010). Drug resistant breast cancer cells overexpress ETS1 gene. Biomed Pharmacother.

[24] Khanna A, Mahalingam K, Chakrabarti D, Periyasamy G (2011). Ets-1 expression and gemcitabine chemoresistance in pancreatic cancer cells. Cell Mol Biol Lett.

[25] Kang HJ, Lee MH, Kang HL, Kim SH, Ahn JR, Na H, Na TY, Kim YN, Seong JK, Lee MO (2014). Differential regulation of estrogen receptor alpha expression in breast cancer cells by metastasis-associated protein 1. Cancer Res.

[26] Yi JS, Mun DG, Lee H, Park JS, Lee JW, Lee JS, Kim SJ, Cho BR, Lee SW, Ko YG (2013). PTRF/cavin-1 is essential for multidrug resistance in cancer cells. J Proteome Res.

[27] Kuscu C, Evensen N, Kim D, Hu YJ, Zucker S, Cao J (2012). Transcriptional and epigenetic regulation of KIAA1199 gene expression in human breast cancer. PLoS One.

[28] Cattoretti G, Andreola S, Clemente C, D'Amato L, Rilke F (1988). Vimentin and p53 expression on epidermal growth factor receptor-positive, oestrogen receptor-negative breast carcinomas. Br J Cancer.

[29] Gruber AD, Pauli BU (1999). Tumorigenicity of human breast cancer is associated with loss of the Ca2+-activated chloride channel CLCA2. Cancer Res.

[30] Walia V, Yu Y, Cao D, Sun M, McLean JR, Hollier BG, Cheng J, Mani SA, Rao K, Premkumar L, Elble RC (2012). Loss of breast epithelial marker hCLCA2 promotes epithelial-to-mesenchymal transition and indicates higher risk of metastasis. Oncogene.

[31] Ishii H, Saitoh M, Sakamoto K, Kondo T, Katoh R, Tanaka S, Motizuki M, Masuyama K, Miyazawa K (2014). Epithelial splicing regulatory proteins 1 (ESRP1) and 2 (ESRP2) suppress cancer cell motility via different mechanisms. J Biol Chem.

[32] Thussbas C, Nahrig J, Streit S, Bange J, Kriner M, Kates R, Ulm K, Kiechle M, Hoefler H, Ullrich A, Harbeck N (2006). FGFR4 Arg388 allele is associated with resistance to adjuvant therapy in primary breast cancer. J Clin Oncol.

[33] Valladares-Ayerbes M, Iglesias-Diaz P, Diaz-Prado S, Ayude D, Medina V, Haz M, Reboredo M, Antolin S, Calvo L, Anton-Aparicio LM (2009). Diagnostic accuracy of small breast epithelial mucin mRNA as a marker for bone marrow micrometastasis in breast cancer: a pilot study. J Cancer Res Clin Oncol.

[34] Yamamoto H, Mukaisho K, Sugihara H, Hattori T, Asano S (2011). Down-regulation of FXYD3 is induced by transforming growth factor-beta signaling via ZEB1/deltaEF1 in human mammary epithelial cells. Biol Pharm Bull.

[35] Tong M, Chan KW, Bao JY, Wong KY, Chen JN, Kwan PS, Tang KH, Fu L, Qin YR, Lok S, Guan XY, Ma S (2012). Rab25 is a tumor suppressor gene with antiangiogenic and anti-invasive activities in esophageal squamous cell carcinoma. Cancer Res.

[36] Pellatt AJ, Wolff RK, John EM, Torres-Mejia G, Hines LM, Baumgartner KB, Giuliano AR, Lundgreen A, Slattery ML (2013). SEPP1 influences breast cancer risk among women with greater native american ancestry: the breast cancer health disparities study. PLoS One.

[37] Turner BC, Zhang J, Gumbs AA, Maher MG, Kaplan L, Carter D, Glazer PM, Hurst HC, Haffty BG, Williams T (1998). Expression of AP-2 transcription factors in human breast cancer correlates with the regulation of multiple growth factor signalling pathways. Cancer Res.

[38] Seksenyan A, Kadavallore A, Walts AE, de la Torre B, Berel D, Strom SP, Aliahmad P, Funari VA, Kaye J (2015). TOX3 is expressed in mammary ER(+) epithelial cells and regulates ER target genes in luminal breast cancer. BMC Cancer.

[39] Cameron EE, Bachman KE, Myohanen S, Herman JG, Baylin SB (1999). Synergy of demethylation and histone deacetylase inhibition in the re-expression of genes silenced in cancer. Nat Genet.

[40] Yamashita Y, Shimada M, Harimoto N, Rikimaru T, Shirabe K, Tanaka S, Sugimachi K (2003). Histone deacetylase inhibitor trichostatin A induces cell-cycle arrest/apoptosis and hepatocyte differentiation in human hepatoma cells. Int J Cancer.

[41] Suzuki T, Yokozaki H, Kuniyasu H, Hayashi K, Naka K, Ono S, Ishikawa T, Tahara E, Yasui W (2000). Effect of trichostatin A on cell growth and expression of cell cycle- and apoptosis-related molecules in human gastric and oral carcinoma cell lines. Int J Cancer.

[42] Roche J, Nasarre P, Gemmill R, Baldys A, Pontis J, Korch C, Guilhot J, Ait-Si-Ali S, Drabkin H (2013). Global Decrease of Histone H3K27 Acetylation in ZEB1-Induced Epithelial to Mesenchymal Transition in Lung Cancer Cells. Cancers (Basel).

[43] Thiery JP, Acloque H, Huang RY, Nieto MA (2009). Epithelial-mesenchymal transitions in development and disease. Cell.

[44] Floor S, van Staveren WC, Larsimont D, Dumont JE, Maenhaut C (2011). Cancer cells in epithelial-to-mesenchymal transition and tumor-propagating-cancer stem cells: distinct, overlapping or same populations. Oncogene.

[45] Brabletz S, Brabletz T (2010). The ZEB/miR-200 feedback loop a motor of cellular plasticity in development and cancer?. EMBO Rep.

[46] Mongroo PS, Rustgi AK (2010). The role of the miR-200 family in epithelial-mesenchymal transition. Cancer Biol Ther.

[47] Wellner U, Schubert J, Burk UC, Schmalhofer O, Zhu F, Sonntag A, Waldvogel B, Vannier C, Darling D, zur Hausen A, Brunton VG, Morton J, Sansom O (2009). The EMT-activator ZEB1 promotes tumorigenicity by repressing stemness-inhibiting microRNAs. Nat Cell Biol.

[48] Lim YY, Wright JA, Attema JL, Gregory PA, Bert AG, Smith E, Thomas D, Lopez AF, Drew PA, Khew-Goodall Y (2013). Epigenetic modulation of the miR-200 family is associated with transition to a breast cancer stem-cell-like state. J Cell Sci.

[49] Zhou X, Wang Y, Shan B, Han J, Zhu H, Lv Y, Fan X, Sang M, Liu XD, Liu W (2015). The downregulation of miR-200c/141 promotes ZEB1/2 expression and gastric cancer progression. Med Oncol.

[50] Cheng JM, Volk L, Janaki DK, Vyakaranam S, Ran S, Rao KA (2010). Tumor suppressor function of Rab25 in triple-negative breast cancer. Int J Cancer.

[51] Xu Y, Gao XD, Lee JH, Huang H, Tan H, Ahn J, Reinke LM, Peter ME, Feng Y, Gius D, Siziopikou KP, Peng J, Xiao X (2014). Cell type-restricted activity of hnRNPM promotes breast cancer metastasis via regulating alternative splicing. Genes Dev.

[52] Vergara-Lluri ME, Moatamed NA, Hong E, Apple SK (2012). High concordance between HercepTest immunohistochemistry and ERBB2 fluorescence in situ hybridization before and after implementation of American Society of Clinical Oncology/College of American Pathology 2007 guidelines. Mod Pathol.

