# Supplementary material for: Epigenetic regulation of ZEB1-RAB25/ESRP1 axis plays a critical role in phenylbutyrate treatment-resistant breast cancer
Source: Oncotarget. 2015 Dec 5;7(2):1741–53. doi: 10.18632/oncotarget.6480 (PMC4811494; doi:10.18632/oncotarget.6480)
Supplement: Supplementary file 1 [file oncotarget-07-1741-s001.pdf]

Epigenetic regulation of ZEB1-RAB25/ESRP1 axis plays a critical role in phenylbutyrate treatment-resistant breast cancer

Supplementary Material

TableS1. PCR sequence of primers and fluorescent probe

method	Gene	Forward primer(5`>3`)	Fluorescent(5`>3`)	Reverse primer(5`>3`)
RT-PCR	β actin	TCACCCACACTGTGCCCATCTACGA		CAGCGGAACCGCTCATTGCCAATGG
RT-PCR	MUCL1	CTGGTACTCTTGGGAGTTTC		TTGGGTAAAACTGGAATGTCT
RT-PCR	RAB25	ATGTTCGCTGAAAACAATGG		GGGTGGACAGATAAAAGAGG
RT-PCR	TFAP2β	GTCCAGCCTATTGTTTGAGA		GTGCCGGTCCTCATAGATA
RT-PCR	ESRP1	AATCGAAATGGCTTATCCCC		GGTTGCATACTGGTAACCTT
RT-PCR/Q-MSP	ANKRD1	AGACAGAGAAGGAGATACCC	AGACTGAACCGCTATAAGATGATCCGA	CACCTATAACCCTGTGCTTT
RT-PCR	AXL	CACTTACAAGACTTGGTCCC		GGGAGCACTGTGATGGT
RT-PCR	CAV1	GTTCGAAGAGGTGGAGTG		TGTAGAGATGTCCCTGCG
RT-PCR/Q-MSP	ETS1	TTTTGGGAAGAAAGTCGGAT	AGCTCCAGATCGACTTTTTCCGTCTTG	CTGCACATTCCATATCCGGG
RT-PCR/Q-MSP	IFI16	TCCTTACTGAGCAACGATTT	CTCTGCTCCTTCAGTTTTGACAGTGC	CTGTTTTCGGGTTCTGAGC
RT-PCR/Q-MSP	KIAA1199	GCATTTTCTTGGTAGCACAA	ATATATCTTTGTTCATGGTGATGCCTACAA	CGAGCAGTGACTTCTTGA
RT-PCR	PTRF	CATGATCTACCAGGATGAAGT	CTTCTCCAGGTTCTCGCGGGTAC	TTTTCCTTGGTCTTGAGGC
Q-MSP	RAB25	TGGGGAATGGAACTGAGGAAGA	TCGCCGATCAGCACCACCTTGAAGACA	CGTGAATCGGGAGAGTAGATTGG
Q-MSP	ESR	GGAGTTCGCCACA	TTTGAATCACCAGGGC	CAGACTTCATCTGG

	P1	GATATTCGTA	CGCCCATCAG	ATAAAGGCATC
RT-PCR /Q-MSP	ZEB1	AACAGTTGGTTTGG TGT	ATCAAGCCAATATTGC ATCC	CTTCACCCATACAA CAAGGT

*1 : RT-PCR was done at 95° C for 3 min followed by 30 cycles at 95° Cfor 1 min, 60° C for 1 min, 72° C for 1 min, and final extension at 72° C for 10 min.

1 μl dNTP mixture, 1.5 μl MgCl2 , 0.2 μmol/l each primer and 0.2 μl Platinum Taq DNA polymerase.

*2 : Bisulfite Sequencing PCR was done based protocol of EZ DNA Methylation-Gold Kit (QIAGEN).

*3 : Q-MSP was done at 95° C for 3 min followed by 40 cycles at 95° C for 20 sec, 60° C for 30 sec, and 72° C for 30 sec, in a 25 μl reaction volume containing 200nmol/l fluorescect probe, and 25 μl iQTM supermix.

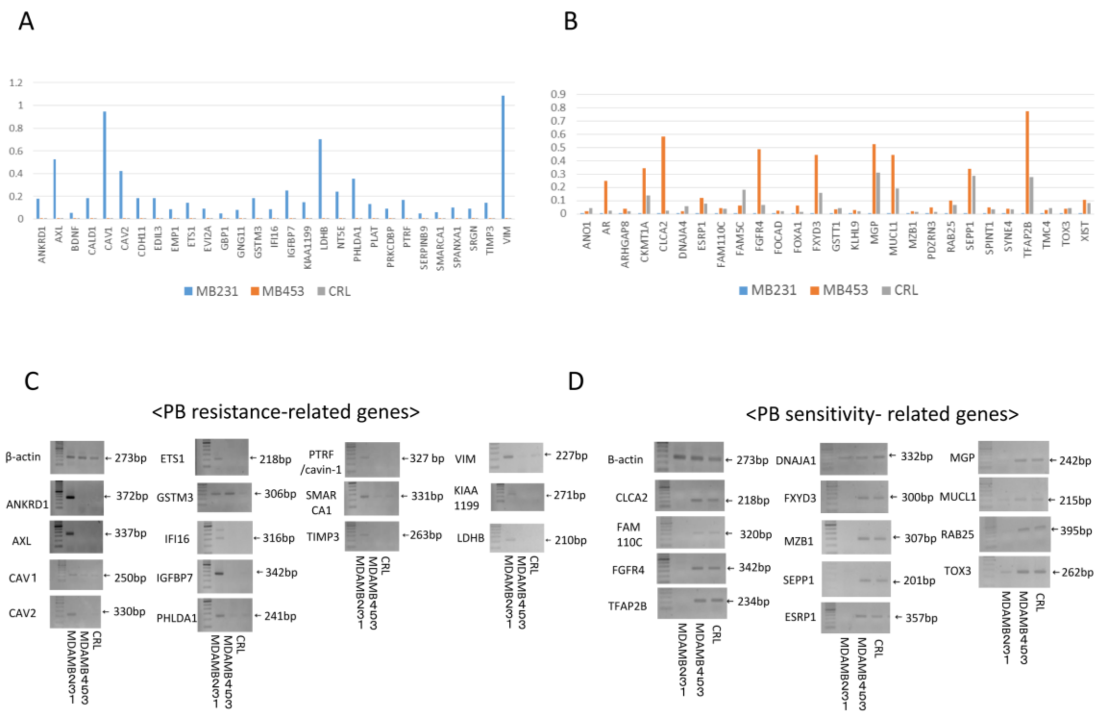

Figure S1. PB resistance-related genes and sensitivity-related genes identified using microarrays.

(A) PB resistance-related genes (those highly expressed in PB-resistant strains and minimally expressed in PB-sensitive strains) identified using microarrays.

(B) PB sensitivity-related genes (those highly expressed in PB-sensitive strains and minimally expressed in PB-resistant strains) identified using microarrays.

(C) mRNA expression levels of PB resistance-related genes in MDAMB453, CRL, and MDAMB231 cells as measured by semi-quantitative RT-PCR.

(D) mRNA expression levels of PB sensitivity-related genes in MDAMB453, CRL, and MDAMB231 cells as measured by semi-quantitative RT-PCR.
